# Effectiveness of Internet-based cognitive-behavioural therapy for obsessive-compulsive disorder (OCD-NET) and body dysmorphic disorder (BDD-NET) in the Swedish public health system using the RE-AIM implementation framework

**DOI:** 10.1016/j.invent.2023.100608

**Published:** 2023-02-15

**Authors:** Lina Lundström, Oskar Flygare, Ekaterina Ivanova, David Mataix-Cols, Jesper Enander, Diana Pascal, Long-Long Chen, Erik Andersson, Christian Rück

**Affiliations:** a Centre for Psychiatry Research, Department of Clinical Neuroscience, Karolinska Institutet, and Stockholm Health Care Services, Region Stockholm, Karolinska University Hospital, SE-141 86 Stockholm, Sweden; b Department of Clinical Neuroscience, Division of Psychology, Nobels väg 9, Karolinska Institutet, 171 77 Stockholm, Sweden; c Centre for Psychiatry Research, Department of Clinical Neuroscience, Karolinska Institutet, SE-113 30 Stockholm, Sweden; d Stockholm Health Care Services, Region Stockholm, CAP Research Centre, Gävlegatan 22, SE-113 30 Stockholm, Sweden

**Keywords:** Obsessive compulsive disorder, Body dysmorphic disorder, Internet-delivered cognitive behaviour therapy, Clinical implementation

## Abstract

**Objectives:**

Therapist-guided internet-delivered cognitive behaviour therapy (ICBT) is an efficacious treatment for obsessive-compulsive disorder (OCD) and body dysmorphic disorder (BDD), but it is unclear if the results obtained in controlled trials can be reproduced in clinical settings. We evaluated the implementation of ICBT for OCD (OCD-NET) and BDD (BDD-NET) in the Swedish public health system.

**Methods:**

Consecutive referrals to an outpatient psychiatric clinic providing ICBT, with a primary diagnosis of OCD or BDD, were included in the study. Four hundred and thirty-four participants started OCD-NET and 163 started BDD-NET. The primary outcome measures were the Yale Brown Obsessive Compulsive Scale (Y-BOCS) and the Y-BOCS for BDD (BDD-YBOCS), respectively. Participants were assessed before treatment, weekly during treatment, and after treatment. The study used the RE-AIM implementation framework, and the elements of reach, effectiveness, adoption, and implementation for the evaluation.

**Results:**

Intention to treat analysis of the OCD-NET sample (*n* = 434) showed a significant decrease in OCD symptoms from pre-treatment to post-treatment (mean reduction = −8.77 [95 % CI -9.48 to −8.05] *p* < .001, *d* = 1.94 [95 % CI 1.75 to 2.13]). Forty-nine percent (95 % CI 43 % to 56 %) of the participants in OCD-NET were classified as treatment responders and 21 % (95 % CI 16 % to 27 %) were in remission. Participants in BDD-NET (*n* = 163) also showed a significant decrease in BDD symptoms from pre-post treatment (mean reduction = −11.37 [95 % CI -12.9 to −9.87] *p* < .001, *d* = 2.07 [95 % CI 1.74 to 2.40]) and 69 % (95 % CI 58 % to 79 %) of the participants were classified as treatment responders and 48 % (95 % CI 38 % to 58 %) were in full or partial remission. Eighty-seven percent of the participants in OCD-NET and 78 % in BDD-NET were treatment completers and participants in both treatment groups reported a high treatment satisfaction at post treatment (OCD-NET = 87 %, BDD-NET = 79 %). The most reported negative effects attributed to the treatments were transient experiences of unpleasant feelings (52 %) and anxiety (50 %). The implementation also influenced treatment delivery and dramatically decreased the mean number of patients waiting to receive face-to-face treatment at the clinic.

**Conclusions:**

Our results indicate that OCD-NET and BDD-NET are suitable treatments for implementation in a Swedish public health service.

## Highlights

• This study evaluated guided Internet-delivered CBT (ICBT) for OCD and BDD when implemented in the Swedish public health system
• Both treatments were associated with significant reductions in OCD and BDD symptom severity at post treatment
• Waiting lists for other treatment options at the clinic were reduced after the implementation of ICBT
• The results suggest that ICBT for OCD and BDD are effective treatments that can be implemented in a Swedish public health service

## 1 Introduction

Obsessive-Compulsive Disorder (OCD) and Body Dysmorphic Disorder (BDD) are closely related psychiatric conditions affecting around 1–2 % of the general population, where women are at greater risk of experiencing OCD and BDD than men 1, 2. Both disorders are associated with functional impairment across multiple life domains, elevated suicide risk 3, 4, and a considerable societal and economic burden 5, 6, 7. OCD and BDD have a low probability of remission if left untreated 8, 9, making access to effective treatment crucial.

Cognitive behavioural therapy (CBT) and medication with selective serotonin reuptake inhibitors (SSRIs) are the recommended first-line treatments for both OCD and BDD [5] but CBT is not readily available in most parts of the world. Common barriers to access treatment include costs associated with treatment [10], geographical distances, a marked shortage of trained CBT therapists [11], shame and stigma. Therapist-guided Internet-delivered cognitive behaviour therapy (ICBT) is a treatment format with identical treatment components as face-to-face CBT, but the online delivery requires less therapist time and enables greater accessibility 12, 13, overcoming some of the traditional barriers to access treatment. To date, a number of clinical trials suggests that ICBT is effective in reducing OCD and BDD symptoms and also cost effective from a societal perspective 14, 15, 16, 17, 18, 19, 20, 21, 22.

OCD-NET, a therapist-guided ICBT program developed for adults with OCD, has shown large effect-sizes in several RCT:s 14, 15, 16 with treatment gains sustained up to two years after treatment [23]. OCD-NET has also been translated into different languages and the English speaking version of the program has been evaluated in one feasibility study, with similar treatment effects as in the Swedish trials [24]. Therapist-guided ICBT for BDD (BDD-NET), developed by the same research group, has been evaluated in one feasibility study [25] and one randomised controlled trial, with positive results [17] and treatment gains maintained up to 2 years after treatment [26]. Results from a second feasibility study with global inclusion, also indicated that BDD-NET can be delivered across international borders to a culturally diverse sample [27]. Importantly, OCD-NET and BDD-NET have mainly been tested in so called “efficacy trials”, i.e. randomised controlled trials, and it cannot be assumed that results obtained under strictly controlled conditions to maximise internal validity, will be extendable to naturalistic clinical settings.

A few research groups have investigated the effectiveness of ICBT for OCD when delivered as part of routine care. Lovell and colleagues investigated the effectiveness of a digital CBT program for OCD, accompanied by limited telephone support. Results showed that the treatment had no significant effect when delivered in a routine service setting, but there were indications that the intervention could reduce the proportion of patients requiring face-to-face CBT [28]. Luu et al. found medium within group effect-size reductions between pre and post treatment (*g* = 0.61) for OCD patients who underwent an online iCBT course (either self-guided or clinician supported) delivered in routine care [29]. Wootton and colleagues evaluated a therapist guided ICBT program for OCD within the MindSpot clinic in Australia. Results indicated that the treatment was associated with meaningful improvements in OCD symptoms (post treatment, *g* = 0.57 and 3-month follow-up, *g* = 0.90), but these effects were considerably smaller than those of previous randomised trials [30]. OCD-NET has been pilot tested in Improving Access to Psychological Therapies (IAPT) services in the UK. Results indicated significant reductions of OCD-symptoms (*d* = 1.77) [31] in the same range as in the published clinical trials 14, 15, 16, 23. Interestingly, the degree of therapist training was found to moderate the treatment results [31].

In summary, research done on ICBT for OCD in regular clinical settings is scarce and effects are generally smaller than what has been obtained in previous efficacy trials 29, 30. To our knowledge, no study has yet examined the effectiveness of ICBT for BDD when implemented in regular health care. Thus, an essential next step is to evaluate if the promising results of OCD-NET and BDD-NET can be generealized to regular health care contexts.

The primary aim of this study was to evaluate the effectiveness of OCD-NET and BDD-NET in the Swedish public health system. Secondary aims investigated elements of adoption, whether OCD-NET and BDD-NET were acceptable regarding treatment uptake and treatment satisfaction. Finally, success of the implementation regarding treatment administration, and if the introduction of ICBT correlated with reductions in the waiting list for regular face-to-face CBT at the clinic, were evaluated.

## 2 Method

This repeated measures study (pre, weekly and post treatment) included all participants enrolled to receive OCD-NET and BDD-NET from the start of the implementation in September 2018 to December 2020. Since OCD-NET and BDD-NET were implemented simultaneously, and because the treatment contents are similar, we decided to evaluate both treatments at the same time. We used the RE-AIM implementation framework for the evaluation, which was developed to aid researchers in the translation of scientific advances into real world settings [32]. RE-AIM evaluates implementation from the dimensions of reach, effectiveness, adoption, implementation and maintenance, and has been extensively used in public health settings [33].

The study took place in a publicly funded outpatient psychiatric clinic in Stockholm, Sweden, providing individual and group face-to-face CBT for OCD-related disorders and ICBT for OCD and BDD. Patients from all parts of Sweden could access the ICBT programs and since healthcare is universal and publicly funded, all Swedish residents had access to treatment without restriction. The study was approved by the Regional Ethics Board of Stockholm (REPN dnr 2018/2550-32) and the methods were pre-registered at Open Science Framework (OSF) before data analysis began, (https://osf.io/rkpgu/).

### 2.1 Participants

All consecutive referrals to the clinic that started OCD-NET or BDD-NET were included in the study, and there were no other inclusion or exclusion criteria related to study participation. The Clinic's guidelines for offering OCD-NET or BDD-NET were that the patient should have a primary diagnosis of OCD or BDD according to DSM-5, be 18 years or older and resident in Sweden. Patients with acute suicidal ideation (present suicidal thoughts/plans), where the need for frequent monitoring was not suitable for the internet format, severe depression (MADRS-S score > 35), current psychotic episode, current substance abuse, reading and/or writing difficulties or insufficient skills in the Swedish language to read and understand the written treatment content were not offered the treatment but were referred to more suitable treatment options. All patients that came to the clinic were informed about the research and consented for their data to be used for research. Patients could opt out from the informed consent, but no one did, and therefore all participant data was included in the current analyses.

### 2.2 Recruitment

**Fig. 1:**
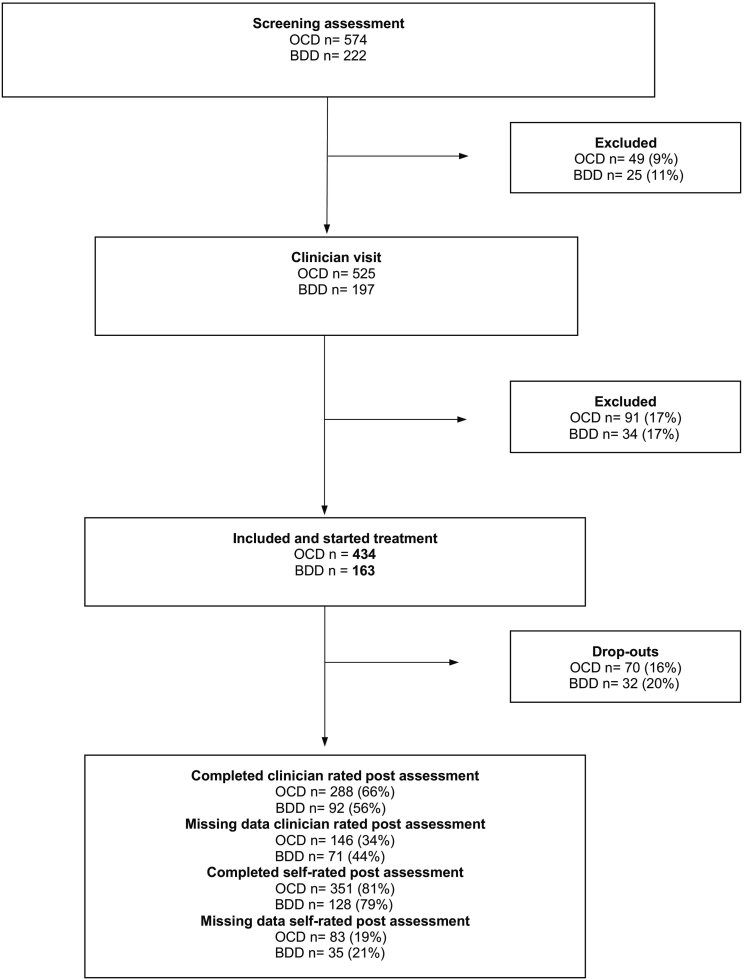
Participant flow through the study. BDD, participants with Body Dysmorphic Disorder; OCD, participants with Obsessive-compulsive disorder; Drop-outs, participants who dropped out of treatment; Missing data; participants who did not provide data at post treatment.

Participants were either clinically referred from a general practitioner (GP) or a psychiatrist or self-referred online by completing an online screening battery at the web page www.internetpsykiatri.se [34]. All participants (both self- and clinically referred) completed a screening and had one or two psychiatric assessments prior to treatment inclusion (Fig. 1). The assessments either took place at the clinic or by encrypted video call. With the outbreak of the covid-19 pandemic in 2020, most of the onsite visits were rescheduled to online video calls. The video assessments were performed in the same way and by the same psychiatrists and psychologists conducting regular face-to-face visits. If deemed eligible for treatment, the participant was enrolled in the study and were given access to OCD-NET or BDD-NET within a week after initial assessment.

### 2.3 Outcome measures according to the RE-AIM elements

The outcome measures for *Reach*, *Effectiveness, Adoption* and *Implementation* are described in Table 1. The primary outcome measure for effectiveness in OCD-NET was the clinician-rated Yale-Brown Obsessive Compulsive Scale (Y-BOCS) [35]. The Y-BOCS measures severity of OCD symptoms and consists of 10 items, divided into the categories of obsessions and compulsion. The total score ranges from 0 to 40 and each item is rated on a 5-point Likert scale ranging from 0 (no symptoms) to 4 (severe symptoms). Y-BOCS has high test-retest reliability (intraclass correlation average = 0.85) and good internal consistency (Cronbach's = 0.87) 35, 36. For BDD-NET, the primary outcome measure was the clinician rated Yale-Brown Obsessive Compulsive Scale for BDD (BDD-YBOCS) [37]. The scale measures BDD symptom severity and consists of 12 items, scored on a 0–4 scale, with a total score ranging between 0 and 48 and a higher score indicating more severe symptoms. The BDD-YBOCS has good psychometric properties with high test–retest reliability (intraclass correlation = 0.83) and high internal consistency (Cronbach's = 0.92) [38] (for a more detailed description of the secondary outcome measures, see eMethod 1 in the online supplement). Treatment response for OCD was defined as Y-BOCS score reduction ≥35 % and CGI-*I* ≤ 2 and remission was defined as Y-BOCS score ≤ 12 and CGI-I ≤ 2 [39]. For BDD, treatment response was defined as BDD-YBOCS score reduction ≥30 % and full or partial remission was defined as BDD-YOCS score ≤ 16 [40].

**Table 1:** Outcome measures for OCD-NET and BDD-NET using the RE-AIM implementation framework.

RE-AIM element	Definition	Metric
Reach	The number and percentage of those invited and eligible who participate and their representativeness	Numbers eligible/excluded and demographic characteristics of included participants
Effectiveness	Change in outcome measures and impact on quality of life and well-being	*OCD-NET*: Yale-Brown Obsessive-Compulsive Scale (Y-BOCS) [35], self-rated Y-BOCS (Y-BOCS-SR) 35, 41, Obsessive Compulsive Inventory—Revised (OCI-R) [42], Montgomery -Åsberg Depression Rating Scale – self-report (MADRS-S) [43], EuroQol 5-dimensions (EQ-5D) [44], treatment response (Y-BOCS score reduction ≥35 % and CGI-*I* ≤ 2) remission (Y-BOCS score ≤ 12 and CGI-I ≤ 2) [39], Negative Effects Questionnaire (NEQ) [45], number of admissions to inpatient psychiatric care during treatment using medical records *BDD-NET*: Yale-Brown Obsessive-Compulsive Scale for BDD (BDD-YBOCS) [37], Appearance Anxiety Inventory (AAI) [46], Montgomery - Åsberg Depression Rating Scale – self-report (MADRS-S) [47], EuroQol 5-dimensions (EQ-5D) [44], treatment response (BDD-YBOCS score reduction ≥30 %) and full or partial remission (BDD-YOCS score ≤ 16) [40], Negative Effects Questionnaire (NEQ) [45], number of admissions to inpatient psychiatric care during treatment using medical records
Adoption	Treatment uptake and satisfaction of the participants	Percentage of participants completing core modules (completers), percentage of participants completing all modules, numbers discontinuing treatment and reasons why, treatment credibility scale (TCS) [48], Client Satisfaction Questionnaire (CSQ) [49]
Implementation and Maintenance	The extent to which a program is delivered consistently, the time and costs, and the long-term effects	Average therapist time spent per participant, number of messages sent during treatment, numbers waiting to receive different treatment options at the clinic and numbers allocated to ICBT

### 2.4 Assessments points

Participants completed an online screening battery after registration and before the psychiatric appointment. If included, self-rated assessments took place at the start of treatment (OCI-R [42], AAI [46], MADRS-S [47], Y-BOCS-SR 35, 41, TCS [48] and EQ-5D) [44], weekly during treatment (OCI-R [42], AAI [46] and MADRS-S) [43], after treatment (OCI-R [42], AAI [46], MADRS-S [47], Y-BOCS-SR [41],EQ-5D [44], CSQ-8 [49], and NEQ) [45] and 6 months after treatment completion (OCI-R [42], AAI [46], MADRS-S [47], self-rated Y-BOCS [41] and EQ-5D) [44]. Clinician rated measures (Y-BOCS [35], BDD Y-BOCS [37] and CGI) [50] were performed by a clinician specialized in OCD and related disorders, before and after treatment.

### 2.5 Interventions

#### 2.5.1 OCD-NET and BDD-NET

OCD-NET consisted of 10 modules and BDD-NET of 8 modules, with text chapters and related work sheets with CBT exercises. Each module included a homework assignment with questions about the text, and modules were unlocked consecutively by the therapist following completion of the homework for each previous module. Participants were encouraged to work with at least one module per week, to have time left if something unexpected happend or if extra time repeating and practicing skills was needed. For a more detailed description of the treatment contents, see previous published studies of OCD-NET and BDD-NET 14, 15, 16, 17, 25 (see also eMethod 2 and 3 in the online supplement).

#### 2.5.2 Therapists

All therapists worked at the clinic and consisted of 10 clinical psychologists specialized in treating OCD-spectrum disorders, 5 practicing psychologists (in their last year of practical training before receiving a psychologist license) and 12 psychology students (at term 6, 8 or 9 out of 10 of their psychology degree). Therapists received external supervision every month and frequently discussed practical and clinical issues related to working with OCD-NET and BDD-NET. Practicing psychologists and psychology students were weekly supervised by a clinical psychologist.

Therapists responded to messages using the Internet platform (8 am-5 pm) on weekdays, to ensure that participants received a response within 24 h. During treatment, therapists encouraged participants to work with the treatment, to do homework assignments and helped them with any problems that occurred. Therapists also monitored participant engagement in treatment and reminded inactive participants to continue to work with the treatment, first through SMS notifications and then through one or two phone calls. If a participant was inactive for >14 consecutive days, despite notifications and telephone contact attempts, treatment was terminated, and the participant was offered a follow-up assessment at the clinic.

### 2.6 Safety and adverse events

During treatment, automatically scheduled self-assessments with MADRS-S were administered weekly in the internet platform and if a participant scored ≥4 on item 9 in MADRS-S (measuring suicidal ideation), their treating psychologist was automatically notified in the platform. The psychologist then contacted the participant by telephone for a structured suicide risk assessment. Before the start of treatment, all participants had to declare contact information regarding their closest emergency psychiatric clinic. If there was an urgent need for psychiatric care according to the structured suicide risk assessment, the participant was scheduled for an immediate face-to-face appointment with a psychiatrist at the clinic or contact was established with the participants closest emergency psychiatric clinic. Adverse events (AEs) were assessed post-treatment with the Negative Effects Questionnaire (NEQ) [45] and by the number of cases admitted to inpatient psychiatric care during treatment.

### 2.7 Statistical analysis

The main outcome analyses were conducted according to the intention to treat principle with the assumption that data was missing at random. For the primary outcome measures (clinician rated Y-BOCS and BDD-YBOCS), linear mixed effects models were used to analyse changes from pre-treatment to post-treatment. The model included fixed effects of time (pre and post) and a random intercept. For the secondary outcome measures, the same mixed effects model was used with fixed effects of time (pre and post for Y-BOCS-SR and EQ-5D and pre, weekly and post for OCI-R, AAI and MADRS-S). Response and remission rates were estimated using generalized linear models, and missing data was imputed using multiple imputation by chained equations. Missingness analyses were performed with unpaired *t*-tests and non-parametric tests for categorical and factor variables (chi-square and Wilcoxon signed-rank test) comparing participants with complete Y-BOCS post data with those missing Y-BOCS post data. Within-group effect sizes were calculated with Cohen's *d* and Alpha for all analyses were set at 0.05.

## 3 Results

### 3.1 Reach

#### 3.1.1 Participant flow and attrition

Participant flow through the study is presented in Fig. 1. Forty-nine (9 %) of the 574 individuals screened for OCD-NET were excluded before the psychiatric appointment and the corresponding number for BDD-NET was 25 individuals (11 %) out of 222. The main reason for exclusion reported by the staff was that the participants did not show up for their psychiatric appointment. For OCD-NET, another 91 (17 %) were excluded after the psychiatric appointment and the corresponding number in BDD-NET was 34 (17 %). Main reasons for exclusion, reported by the clinicians, were that OCD/BDD was not the primary diagnosis or that the participant had severe depression with current suicidal ideation.

Four hundred and thirty-four participants started OCD-NET, and 163 participants started BDD-NET and were included in the study. Their demographic and clinical characteristics are presented in Table 1. For OCD-NET, 66 % completed the clinician rated Y-BOCS post assessment and 34 % had missing data (Fig. 1). For BDD-NET, 56 % completed the BDD-YBOCS assessment at post-treatment and 44 % had missing data. The self-rated post-treatment assessments were administered in the internet platform and therefore the completion rate was higher for the self-rated measures: 81 % in OCD-NET and 79 % in BDD-NET. In OCD-NET, 70 participants dropped out of treatment and in BDD-NET the corresponding number was 32 participants (see Section 3.3 for a more detailed description of drop-outs).

#### 3.1.2 Missingness analysis

The Little's test for missing data was calculated for Y-BOCS and BDD-YBOCS from pre to post treatment. For Y-BOCS, the test was not statistically significant (*n* = 434; *p* = .74), supporting the assumption that data was missing completely at random. For BDD-YBOCS, the test was significant (*n* = 163; *p* < .001), indicating that data was not missing completely at random. Missingness analysis of the participants who completed the clinician rated Y-BOCS post assessment showed that these participants did not differ from those who did not complete this measure on the baseline variables: pre symptom score (Y-BOCS, MADRS-S), age, gender, comorbidity, age onset for OCD and educational level, all (*p* > .05). Missingness analysis of the participants who completed the clinician rated BDD-YBOCS assessment showed no significant differences between those who did not complete this measure on the same baseline variables: pre symptom score (BDD-YBOCS, MADRS-S), age, comorbidity and educational level (*p* > .05), but there were significant differences for age onset for BDD and gender, where men had a higher proportion of missing data than women (69% v.s 37 %) (*p* < .05), eTable 2 in the online supplement.

#### 3.1.3 Participant characteristics

In OCD-NET, 63 % of the sample were women, the mean participant age was 31.4 (9.36) years and the mean number of years since OCD diagnosis was 9.9 (10.02) years (Table 2). The most common psychotropic medication was SSRIs (25 %) and psychiatric comorbidity was prevalent, where the most reported comorbid diagnosis was depression (15 %). In BDD-NET, 79 % of the sample were women, the mean participant age was 30.5 (9.73) years and the mean number of years since BDD diagnosis was 11.10 (9.26) years. The most common psychotropic medication was SSRIs (66 %) and the most reported comorbid diagnosis was depression (31 %).

**Table 2:** Participant characteristics at baseline.

Demographics	OCD-NET	BDD-NET
Source of referral, N (%)	OCD = N/434	BDD = N/163
Clinical referral	79 (18)	12 (7)
Self-referral	355 (82)	151 (93)
Age, mean (SD)	31.4 (9.36)	30.5 (9.73)
Gender, N (%)	OCD = N/434	BDD = N/163
Female	273 (63)	128 (79)
Male	161 (37)	35 (21)
Years with OCD/BDD diagnosis, mean (SD)	OCD = N/362	BDD = N/129
	9.9 (10.02)	11.1 (9.26)
Previous CBT treatment for OCD/BDD, N (%)	OCD = N/406	BDD = N/153
	140 (34)	28 (18)
Previous suicide attempts, mean (SD)	OCD = N/276	BDD ***N*** = 101
	0.15 (0.49)	0.27 (0.69)
Source of income, N (%)	OCD = N/400	BDD=N/156
Employed	261 (65)	89 (57)
Student	87 (22)	50 (32)
On sick leave	27 (7)	11 (7)
Unemployed	19 (5)	5 (3)
Retired	6 (1)	1 (1)
Level of education, N (%)	OCD = N/418	BDD = N/161
Primary school	40 (10)	25 (16)
High school	170 (41)	70 (43)
Vocational school	19 (5)	4 (2)
College/university	189 (45)	62 (39)
Main obsessions, N (%)^a^	OCD = N/406	
Aggressive	236 (58)	N/A
Contamination	194 (48)	N/A
Unacceptable thoughts	129 (32)	N/A
Symmetry	62 (15)	N/A
Main compulsions, N (%)^a^	OCD = N/406	N/A
Checking	291 (72)	N/A
Washing	180 (44)	N/A
Mental rituals	145 (36)	N/A
Ordering	110 (27)	N/A
Most common body areas of concern, N (%)^b^		BDD = N/119
Skin	N/A	52 (44)
Face	N/A	45 (38)
Hair	N/A	37 (31)
Waist/belly	N/A	30 (25)
Eyes	N/A	25 (21)
Current medications, N (%)^c^	OCD = N/133	BDD = N/61
SSRI	95 (25)	40 (66)
Sleep medication	30 (8)	8 (14)
Other antidepressant	18 (5)	12 (20)
Anti-anxiety medication	17 (5)	4 (7)
Anti-psychotic	16 (4)	7 (11)
Antihistamine	15 (4)	3 (5)
Central stimulants	13 (3)	3 (5)
Psychiatric comorbidities, N (%)	OCD = N/406	BDD = N/153
Depression	59 (15)	47 (31)
Generalized anxiety disorder	58 (14)	14 (9)
Social anxiety disorder	18 (5)	23 (15)
ADHD	15 (4)	7 (5)
Autism spectrum disorder	13 (3)	4 (3)
Panic disorder	9 (2)	3 (2)
OCD/BDD	9 (2)	7 (5)
Excoriation disorder	7 (2)	3 (2)
Health anxiety disorder	6 (1)	1 (1)
Post-traumatic stress disorder	5 (1)	1 (1)
Bipolar disorder	4 (1)	2 (1)
Tic disorder	2 (1)	1 (1)
Trichotillomania	1 (1)	0 (0)
Eating disorder	1 (1)	4 (3)

Abbreviations: ADHD, Attention-deficit hyperactivity disorder; BDD, Body Dysmorphic Disorder; CBT, Cognitive behaviour therapy; OCD, Obsessive-compulsive disorder; SSRI, Selective serotonin reuptake inhibitor; N/A, not applicable.

a Participants could report more than one category of obsessions and compulsions.

b Participants could report more than one body area of concern.

c Participants could report more than one medication.

### 3.2 Effectiveness

#### 3.2.1 Primary outcome – clinician-rated measures

**Fig. 2:**
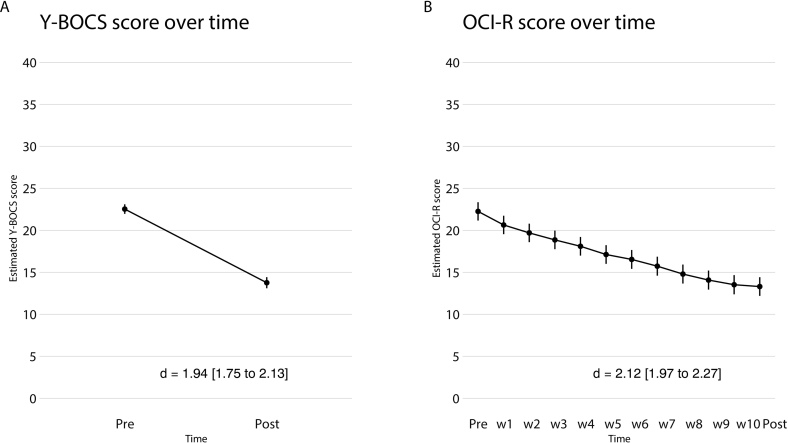
A: Mean Y-BOCS score over time (pre-post) B: Mean OCI-R score over time (pre, weekly, post). Y-BOCS, Yale- Brown Obsessive-Compulsive Scale; OCI-R, Obsessive-compulsive Inventory – Revised; d, Cohen's *d* with-in group effect size.

Analysis of the ITT sample (*n* = 434) showed a significant decrease in OCD symptoms from pre-treatment to post-treatment according to the clinician rated Y-BOCS (mean reduction = −8.77, SE = 0.37, [95 % CI -9.48 to −8.05], *p* < .001) (Fig. 2A) with a large within-group effect size at post treatment (*d* = 1.94 [95 % CI 1.75 to 2.13]). At post-treatment, response rates were 49 % (95 % CI 43 % to 56 %) and remission rates were 21 % (95 % CI 16 % to 27 %).

**Fig. 3:**
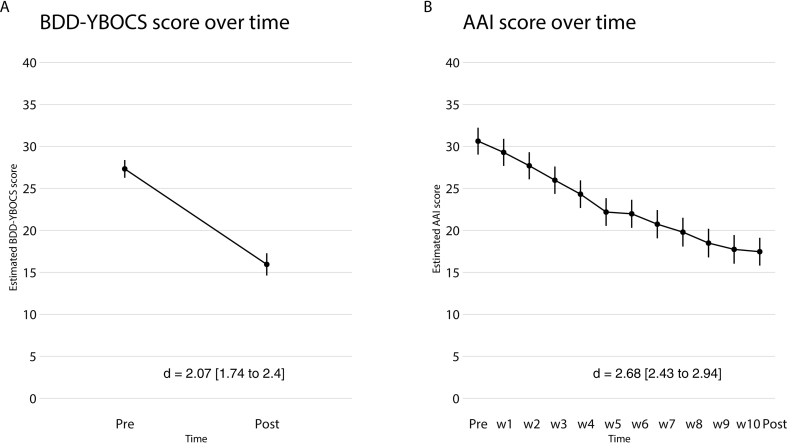
A: Mean BDD Y-BOCS score over time (pre-post) B: Mean AAI score over time (pre, weekly, post). BDD-YBOCS, Yale- Brown Obsessive-Compulsive Scale for BDD; AAI, Appearance Anxiety Inventory; d, Cohen's *d* with-in group effect size.

Analysis of the ITT sample (*n* = 163) also showed a significant decrease in BDD symptoms from pre-treatment to post-treatment according to the clinician rated BDD-YBOCS (mean reduction = −11.37, SE = 0.76, [95 % CI -12.9 to −9.87], *p* < .001) (Fig. 3A) with a large within-group effect size at post treatment (*d* = 2.07 [95 % CI 1.74 to 2.4]). The response rate at post-treatment was 69 % (95 % CI 58 % to 79 %) and the full or partial remission rate was 48 % (95 % CI 38 % to 58 %).

#### 3.2.2 Secondary outcomes – self-rated measures

There was a significant decrease in Y-BOCS-SR scores from pre-treatment to post treatment (mean reduction = −6.13, SE = 0.32, [95 % CI -6.76 to −5.51], *p* < .001), with large effect sizes at post treatment (*d* = 1.43 [95 % 1.26 to 1.59]). Scores on the OCI-R (OCD-NET) and AAI (BDD-NET) also decreased significantly from pre to post treatment (OCI-R mean reduction = −8.95, SE = 0.31, [95 % CI -9.56 to −8.34], *p* < .001; AAI mean reduction = −13.16, SE = 0.59, [95 % CI -14.3 to −12.0], *p* < .001). For all other secondary outcome measures, there were significant reductions at post treatment (see Table 3A, Table 3B, Figs. 2B and 3B and eTable 3 in the online supplement).

**Table 3A:** Results for primary and secondary outcome measures in OCD-NET.

Outcome	Mean and (SE)	Mean reduction (pre-post) and *p*-value	Within-group effect sizes Cohen's *d* (95 % CI)
Y-BOCS			
Pre	22.53 (0.29)		
Post	13.76 (0.34)	−8.77 (<0.001)	1.94 (1.75–2.13)

Y-BOCS-SR			
Pre	21.44 (0.29)		
Post	15.30 (0.32)	−6.13 (<0.001)	1.43 (1.26–1.59)

OCI-R			
Pre	22.25 (0.56)		
Post	13.35 (0.57)	−8.95 (<0.001)	2.12 (1.97–2.27)

MADRS-S			
Pre	16.65 (0.43)		
Post	11.77 (0.44)	−4.88 (<0.001)	1.26 (1.11–1.41)

EQ-5D			
Pre	0.52 (0.02)		
Post	0.64 (0.02)	0.12 (<0.001)	0.44 (0.59–0.30)

Abbreviations: CI, confidence interval; EQ-5D, EuroQol 5-dimensions; MADRS-S, Montgomery-Åsberg Depression Rating Scale Self-Rated; OCI-R, Obsessive-compulsive Inventory – Revised; Y-BOCS, Yale- Brown Obsessive-Compulsive Scale; Y-BOCS-SR, Yale- Brown Obsessive-Compulsive Scale – Self-Rated.

**Table 3B:** Results for primary and secondary outcome measures in and BDD-NET.

Outcome	Mean and (SE)	Mean reduction (pre-post) and p-value	Within-group effect sizes Cohen's *d* (95 % CI)
BDD Y-BOCS			
Pre	27.31 (0.54)		
Post	15.94 (0.67)	−11.37 (<0.001)	2.07 (1.74–2.4)

AAI			
Pre	30.61 (0.81)		
Post	17.45 (0.84)	−13.16 (<0.001)	2.68 (2.43–2.94)

MADRS-S			
Pre	19.02 (0.73)		
Post	12.64 (0.75)	−6.38 (<0.001)	1.48 (1.24–1.73)

EQ-5D			
Pre	0.50 (0.02)		
Post	0.65 (0.03)	0.15 (<0.001)	0.57 (0.81–0.33)

Abbreviations: CI, confidence interval; AAI, Appearance Anxiety Inventory; BDD-YBOCS, Yale- Brown Obsessive-Compulsive Scale for BDD; EQ-5D, EuroQol 5-dimensions; MADRS-S, Montgomery-Åsberg Depression Rating Scale Self-Rated.

#### 3.2.3 Adverse events

The most common negative effects rated by the participants and attributed to the treatments were experiences of more anxiety (OCD-NET = 52 %, BDD-NET = 52 %) and unpleasant feelings (OCD-NET = 50 %, BDD-NET = 50 %), which are expected from an ERP treatment and thought to subside after treatment (eTable 5, online supplement). In OCD-NET, 5 participants were admitted to in-patient care during treatment due to worsening of symptoms related to OCD or depressive mood. No participants in BDD-NET were admitted to in-patient care during treatment and no serious adverse events (i.e., self-harm or suicide attempts/suicide) occurred in either group.

### 3.3 Adoption

#### 3.3.1 Treatment completers and drop-outs

Participants in OCD-NET completed a mean number of 8 (SD = 3.01) modules and 264 (61 %) of the participants completed all 10 modules. Three hundred and seventy-nine (87 %) of the participants were treatment completers, meaning that they were assigned at least 4 modules and started with ERP. Seventy of the participants (16 %) in OCD-NET discontinued treatment beforehand and the main reason for this was inactivity (*n* = 25) in the internet platform (if a participant was inactive for >14 days without responding to contact attempts from the therapist, he/she was cancelled from the treatment). Other reasons for discontinuation were: not enough time to work with the treatment (*n* = 17), the internet format was not suitable (*n* = 14), worsening of symptoms and not able to continue with the treatment (*n* = 10), main diagnosis was no longer OCD (*n* = 2), or so much improvement that the participant did not want to finish the treatment (n = 2).

For participants in BDD-NET, the mean number of modules completed was 6.5 (SD = 2.23) and 100 (61 %) of the participant completed all 8 modules. One-hundred and twenty-seven (78 %) of the participants were treatment completers, meaning that they were assigned at least 5 modules and started with ERP. Thirty-two (20 %) of the participants in BDD-NET discontinued treatment beforehand and the main reason for this was inactivity (*n* = 20) in the internet platform. Other reasons for discontinuation were: the internet format was not suitable (*n* = 3), main diagnosis was no longer BDD (n = 3), so much improvement that the participant did not want to finish the treatment (n = 3), not enough time to work with the treatment (n = 2), or worsening of symptoms and not able to continue with the treatment (*n* = 1).

#### 3.3.2 Treatment satisfaction and credibility

For participants in OCD-NET, the mean score on CSQ-8 was 26.5 (SD = 4.15), indicating a high degree of satisfaction. Eighty-seven percent of the participants reported that they were mostly to very satisfied with ICBT according to item 7 on CSQ-8 and 90 % assessed the treatment quality as good or excellent (item 1). For participants in BDD-NET, the mean score on CSQ-8 was 25.2 (SD = 4.14), and 79 % of the participants reported that they were mostly to very satisfied with ICBT and 90 % assessed the treatment quality as good or excellent. Participants in both OCD-NET and BDD-NET reported a high treatment credibility and expectation of improvement at week 3 (OCD-NET; C-scale mean = 35.9 [SD = 7.60], BDD-NET; C-scale mean = 33.8 [SD = 8.07]).

### 3.4 Implementation and maintenance

The ICBT platform logged the number of messages and amount of time that therapists spent interacting with participants (e.g., reviewing homework assignments and writing e-mails). In OCD-NET, therapists sent on average 23.6 messages (*SD* = 17.96) over the course of treatment (12 weeks), and the average therapist time spent per participant during the entire treatment period was 109.9 min (*SD* = 126.79), corresponding to an average of 9.2 (SD = 10.57) minutes of therapist time per participant and week. Therapists in BDD-NET sent on average 20 messages (*SD* = 12.73) over the course of treatment (12 weeks), and the average therapist time spent per participant in BDD-NET during the entire treatment period was 86.3 min (*SD* = 105.02), corresponding to an average of 7.2 (SD = 8.75) minutes of therapist time per participant and week.

#### 3.4.1 Average number of patients on the waiting list to receive different treatment options at the clinic

To investigate the impact of the implementation of OCD-NET and BDD-NET on clinic variables, data on the number of people waiting to receive different treatment options at the clinic and the number of people starting internet treatment, was collected between 2018 and 2020. The implementation started in September 2018 and during 2019, ICBT could be considered an established treatment option at the clinic.

**Fig. 4:**
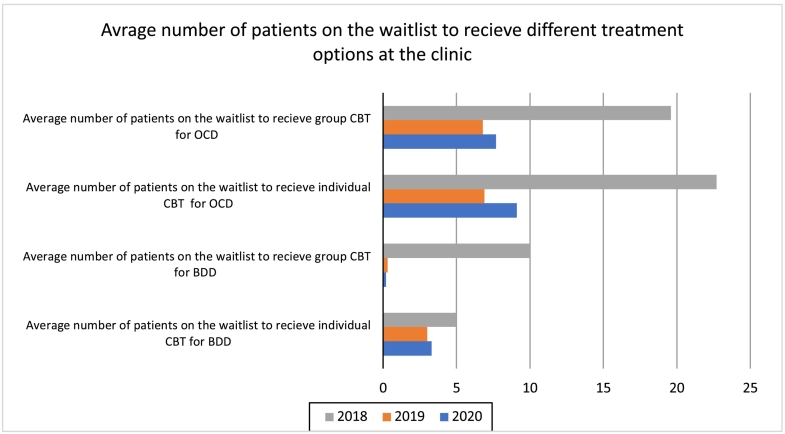
Effect of OCD-NET and BDD-NET on waiting lists at the clinic. BDD, Body Dysmorphic Disorder; OCD, Obsessive-compulsive disorder; CBT, Cognitive behaviour therapy.

In 2018, the average number of patients on the waiting list for group CBT for OCD was 19.6 patients (see Fig. 4). In 2019, this number decreased to 6.8 patients, a reduction of 65 %. The pattern continued in 2020, when the average number on the waiting list was 7.7 patients, a reduction of 61 % compared to 2018. Similarly, the average waiting list for individual CBT decreased from 22.7 patients in 2018 to 6.9 patients in 2019, corresponding to a 70 % reduction. The numbers waiting to receive group and individual CBT for BDD during 2018–2020 followed a similar pattern as for OCD (Fig. 4). Of note, the decrease in waiting lists for regular CBT was coupled with no waiting time for OCD-NET and BDD-NET, which always started within a week after inclusion.

#### 3.4.2 Number of participants starting ICBT

For OCD-NET, 27 patients were allocated to ICBT and started treatment in 2018, 193 in 2019 and 214 in 2020. For BDD-NET, the corresponding number was 6 patients 2018, 70 in 2019 and 87 in 2020. At the end of the implementation phase, all psychologists at the clinic treated ICBT participants as part of their regular caseloads.

#### 3.4.3 6-month follow-up

The number of participants providing data for the 6-month follow-up assessment was 112 (26 %) in OCD-NET and 34 (21 %) in BDD-NET. With 74 % data-loss in OCD-NET and 79 % in BDD-NET, the planned 6-month follow-up analysis could therefore not be performed.

## 4 Discussion

This study evaluated the implementation of ICBT for OCD and BDD in the Swedish public health system. Both treatments were associated with significant reductions in OCD and BDD symptom severity at post treatment (mean Y-BOCS reduction = −8.8 points, mean BDD-YBOCS reduction = −11.4 points). Forty-nine percent of the participants in OCD-NET and 69 % of the participants in BDD-NET responded to treatment and both treatments led to large effect sizes at post treatment (OCD-NET, *d* = 1.94; BDD-NET, *d* = 2.07). The treatments were also associated with improvements on secondary outcomes (mean OCI-R reduction = −8.9, mean AAI reduction = −13.16 and mean MADRS-S reduction = OCD-NET: -4.88, BDD-NET: −6.38). A high proportion of participants were women and the most common psychiatric comorbidities were depression and anxiety disorders, which echo results from prevalence studies on OCD and BDD 1, 2, 51. A majority of the participants were employed and only 7 % in each of the samples were on sick-leave, which may be less representative for the societal and economic burdens that are commonly described in OCD and BDD 1, 2. A high percentage of participants in OCD-NET (87 %) and BDD-NET (79 %) were satisfied with the treatment and module completion was high in both treatment groups (OCD-NET, mean 8 modules; BDD-NET, mean 6.5 modules). Further, waiting lists for other OCD and BDD treatment options at the clinic were reduced by 60–70 % after the implementation of ICBT. Experiences of transient increases in anxiety and unpleasant feelings related to the treatment were the most common negative effects reported by the participants in both treatments. A main treatment component of ERP is learning to tolerate increases in anxiety and unpleasant feelings, so these negative effects were expected [52].

Studies examining the effectiveness of ICBT for OCD in clinical settings have found medium within group effect sizes of around 0.6 28, 29, 30, which is considerable smaller than the large effects found in previous RCT studies of ICBT for OCD (*d* = 0.87 to 1.55) 14, 18, 19. In the current study, the within group effect sizes (OCD-NET *d* = 1.94 and BDD-NET *d* = 2.07) were on par with the large within group effect sizes observed in previous RCT:s for OCD-NET (*d* = 1.25 to 2.20) 14, 15, 16 and BDD-NET (*d* = 1.42) [17] and similar to the effectiveness results (*d* = 1.77) of OCD-NET when implemented within the IAPT system in England [31]. To our knowledge, this is the first study evaluating the effectiveness of ICBT for BDD as part of routine care. The results are promising and in line with the large effects seen in previous studies of BDD-NET performed in research settings 17, 25, 27.

Eighty-seven percent of the participants in OCD-NET and 78 % in BDD-NET were classified as treatment completers and 49 % (OCD-NET) and 69 % (BDD-NET) responded to treatment. The response and completer rates in the current study are higher than what has been observed in previous routine care studies of ICBT for OCD 28, 29, 30 and higher than the only RCT study of BDD-NET [17]. In OCD-NET and BDD-NET, the therapists are instructed to respond with short but frequent messages within 24 h. One possibility is that the frequent therapist contact contributed to the higher number of treatment completers in this study, compared to other digital ICBT programs with less frequent therapist support. The therapists in this study only spent 9.2 mi (OCD-NET) and 7.2 min (BDD-NET) per participant per week delivering treatment, meaning that the frequent therapist contact did not interfere with an efficient provision of therapy. These results also confirm those of previous health economic analyses conducted alongside the efficacy clinical trials, indicating that the minimal therapist time, make these treatments highly cost-effective 16, 21, 53.

One important additional finding in this study was that ICBT was associated with a significant decrease in the average number of patients waiting to receive face-to-face treatment options at the clinic. This effect was seen within a few months after implementation, and the number of participants receiving ICBT increased over the course of the three years. All psychologists at the clinic started working with ICBT, and since the therapist time for ICBT was minimal, the clinic could scale up their treatment delivery, leading to fewer people waiting for treatment and to more people receiving help in a timely manner, one of the main goals of ICBT [12]. All these transformations can be seen as indicators for a successful implementation at the clinic and an effective way to reach out to the target population.

This study was carried out in a clinical setting with procedures and staff that were part of routine care. The naturalistic setting and broader guidelines for inclusion and exclusion compared to previous efficacy studies on OCD-NET and BDD-NET, increase the external validity and generalisability of the results. The study used clinician rated gold standard outcome measures (Y-BOCS and BDD-YBOCS), which many previous effectiveness studies of OCD lacked 29, 30, 31.This study used the RE-AIM implementation framework to evaluate the implementation from not only an effectiveness perspective, but also from the elements of reach, adoption,implementation and maintenance. By including additional evaluation criteria, this study aimed at capturing more aspects of the implementation process, thus adding new findings to the existing literature of effectiveness trials in OCD and BDD.

The study also had some limitations, which are largely inherent to the naturalistic design. First, there was no control group meaning that the observed effects could be attributed to other factors than the treatments themselves e.g. passage of time, therapeutic attention or the natural fluctuation of symptoms. However, spontaneous recovery is rarely seen in OCD [54] and BDD [9] and the treatment effects in this study are comparable to those observed in previous studies of ICBT for OCD and BDD. Second, the naturalistic setting contributed to more data loss and drop-outs than in previous randomised trials testing OCD-NET and BDD-NET, which represents a threat to the internal validity of the study. However, the data attrition in this current study is in line with previous studies on ICBT for OCD conducted in regular health care settings 29, 30, 31. Additionally, the effects observed from the self-rated measures (with less data loss) were in line with the effects captured by the clinician rated outcome measures. No data attrition bias effects were seen in the missingness analysis for OCD-NET, while significant differences were found for age onset and gender in BDD-NET, meaning that these results should be interpreted more cautiously. Third, this study did not use blinded assessors or perform inter-rater reliability data checks from the assessing clinicians. However, it is important to stress that the assessors involved in this study were experts in OCD-related disorders and many of them have also been assessors in previous clinical trials of OCD-NET and BDD-NET, where reliability checks have been performed [16]. Although the treatments were delivered as part of routine care, the study was still conducted in a specialized psychiatric unit, with therapists trained to treat patients with OCD-related disorders. The treatment effects might therefore not be equally generalizable to other treatment contexts, such as primary care. Further, participants may not be representative for the broader OCD and BDD population, although all patients deemed eligible for ICBT were included in the study. We planned an analysis of the 6-month follow-up outcomes, but unfortunately data-loss was extensive, so no conclusions regarding maintenance of the effectiveness results can be drawn.

## 5 Conclusion

The results from this study suggest that OCD-NET and BDD-NET are effective in treating symptoms of OCD and BDD, acceptable for patients, and safe when delivered by trained therapists in a specialized psychiatry unit. The implementation shortened waiting times to face-to-face treatment and during the course of the study, internet delivered treatments became a significant part of the clinic's regular activity. Altogether, the findings indicate that guided ICBT for OCD and BDD are suitable treatments for implementation in the Swedish public health system.

## CRediT authorship contribution statement

*Concept and design*: Lundström, Rück, Andersson, Mataix-Cols.

*Acquisition, analysis, or interpretation of data*: Lundström, Flygare, Andersson, Ivanova, Enander, Pascal, Chen, Mataix-Cols, Rück.

*Drafting of the manuscript*: Lundström, Andersson, Mataix-Cols, Rück.

Critical revision of the manuscript for important intellectual content: All authors.

*Statistical analysis*: Lundström, Ivanova, Flygare, Andersson.

*Obtained funding*: Flygare, Andersson, Rück.

Administrative, technical, or material support: Andersson, Enander, Rück.

*Supervision*: Andersson, Ivanova, Mataix-Cols, Rück.

## Declaration of competing interest

The authors declare the following financial interests/personal relationships which may be considered as potential competing interests:

Prof. Mataix-Cols receives royalties for contributing articles to UpToDate, Wolters Kluwer Health, outside of the submitted work.

Prof. Rück has received book royalties from Natur & Kultur, Studentlitteratur and Albert Bonniers Förlag and various speakers fees all unrelated to the submitted work.

Dr Andersson receives royalties from Natur & Kultur for a self-help book on health anxiety

Dr Flygare has received speaking fees from the Swedish OCD Association, Insight Events AB, and Kry International AB, as well as reimbursement for writing articles for Inside Practice Psychiatry, all outside the submitted work.

Dr Enander has no competing interests to declare.

Dr Radu has no competing interests to declare.

Dr Ivanova has no competing interests to declare.

MD Chen has no competing interests to declare.

## Appendix A Supplementary data

Supplementary material Image 1
