# Supplementary material for: Effectiveness of Internet-based cognitive-behavioural therapy for obsessive-compulsive disorder (OCD-NET) and body dysmorphic disorder (BDD-NET) in the Swedish public health system using the RE-AIM implementation framework
Source: Internet Interv. 2023 Feb 15;31:100608. doi: 10.1016/j.invent.2023.100608 (PMC9958485; doi:10.1016/j.invent.2023.100608)
Supplement: Supplementary file 1 — Supplementary material [file mmc1.docx]

# **Supplemental Online Content**

**eMethod 1.** Description of secondary outcome measures

**eMethod 2.** OCD-NET module treatment content

**eMethod 3.** BDD-NET module treatment content

**eFigure 1.** Online Treatment Platform, Homework view

**eTable 1.** Body areas of concern, BDD-NET

**eTable 2.** Results from missingness analysis

**eTable 3.** Weekly results for secondary outcome measures

**eTable 4.** Results for the complete case analysis of response/remission

**eTable 5.** Results from the Negative effects questionnaire (NEQ)

**eReferences**

**eMethod 1. Description of secondary outcome measures**

**The Yale Brown Obsessive Compulsive Scale - Self Report (Y-BOCS-SR)**
The Yale Brown Obsessive Compulsive Scale – Self Report (Y-BOCS-SR) is a self-rated scale for measuring severity of OCD symptoms. The scale consist of 10 items divided into the categories of obsessions and compulsion, rated on a 5-point Likert scale ranging from 0 (no symptoms) to 4 (severe symptoms). The total score ranges from 0 to 40. Y-BOCS-SR has high test-retest reliability (intraclass correlation average= 0.85) and good internal consistency (Cronbach’s = 0.87).^1,2^

**Obsessive Compulsive Inventory - Revised (OCI-R)**

The Obsessive Compulsive Inventory - Revised (OCI-R) is an 18 item self-report measure of OCD severity. It measures six different symptom dimensions of OCD and each item is scored on a scale from 0-4, with a total score of 72. A higher score indicates more severe OCD. The OCI–R has excellent psychometric properties, and the subscales differentiate well between individuals with and without OCD.^3,4^

**The Appearance Anxiety Inventory (AAI)**

The Appearance Anxiety Inventory (AAI) is a 10 item self-report scale measuring cognitive processes, threat monitoring and avoidance behaviours related to symptoms of BDD. The items are scored on a 5-point Likert scale ranging from 0 (not at all) to 4 (all the time), with a total score of 0-40. The AAI has shown high internal consistency (Cronbach’s  = 0.86) and test–retest reliability (intraclass correlation = 0.87).^5^

**Montgomery Åsberg Depression Rating Scale - Self report (MADRS-S)**

The Montgomery Åsberg Depression Rating Scale-Self report **(**MADRS-S) is a self-report measure of depression severity, with a total score ranging from 0 to 54, where a higher score indicates more severe depression. The scale consists of nine items, each measuring a different symptom on a seven-point scale. The MADRS-S has good to excellent test–retest reliability (r=0.80–0.94).^6^

**The Euroqol 5 dimensions (EQ-5D)**

The euroqol 5 dimensions (EQ-5D) is a self-reported generic measurement of global functioning and quality of life. It measures five health domains of importance to quality of life and each domain is scored from 0-3 where 0 indicates no problems and 3 indicates extreme problems. EQ-5D has good test-retest reliability (intraclass coefficient = 0.82) and acceptable convergent validity.^7^

**The Clinical Global Impression (CGI)**

The Clinical Global Impression (CGI) is a clinician-rated measure of clinical global severity of illness (CGI-S) and clinical global improvement (CGI-I). The CGI-S scores range from 1 (not at all ill) to 7 (extremely ill), and the CGI-I scores range from 1 (very much improved) to 7 (very much worse).^8^ CGI has shown good reliability and validity for a range of psychiatric disorders.^9,10^

**The Credibility Expectancy Questionnaire**

The Credibility Expectancy Questionnaire measures treatment credibility and expectancy for improvement. It is a self-report six item questionnaire where Items 1 to 3 and 5 are answered on a scale ranging from 1 (not at all) to 9 (very much) and items 4 and 6 are answered on a 0 (not at all) to 100% (very much) range. The questionnaire demonstrates high internal consistency within each factor and good test-retest reliability.^11^

**The Client Satisfaction Questionnaire (CSQ-8)**

The Client Satisfaction Questionnaire (CSQ) is a self-report 8-item measure for assessing treatment satisfaction with a specific healthcare or counseling service. The score ranges from 8 to 32, with higher scores indicating greater satisfaction with a service. The 8-item version of the scale has a Cronbach's alpha of 0.92-0.93.^12^

**The Negative Effects Questionnaire (NEQ)**

The Negative Events Questionnaire (NEQ) is a 32 item self-rated measure of negative events as perceived by the participants as caused by the treatment or by other factors. The NEQ generates frequencies of negative events and their negative impact on a scale from 0 (not at all) to 4 (extreme). The NEQ has a high internal consistency with a Cronbach's alpha of 0.95.^13^

**eMethod 2.**

|  | **OCD-NET: Description of module content and number of participants completing each module** |  |
| --- | --- | --- |
| 1 | **Psychoeducation:** Introduction to the treatment and information about OCD, common symptoms, prevalence, and main principles of conducting an online CBT treatment. Different fictional patient characters are introduced and used as examples to help clarify the treatment components throughout the treatment. Participants begin to register OCD-related behaviors and thoughts in an online diary | 23 (5%) |
| 2 | **A cognitive–behavioural conceptualisation of OCD:** Participants begin to link obsessions and compulsions to the OCD circle and learn how to conduct a functional analysis of their OCD problems. Participants continue to register OCD-related thoughts and behaviours and apply these to the OCD circle*.* | 16 (4%) |
| 3 | **Cognitive restructuring:** Common OCD metacognitions are explained, such as inflated responsibility, absolute need for certainty, thought-action fusion and exaggerated need to control. Participants use their diary registrations to analyse meta cognitions associated with obsessions. | 15 (3%) |
| 4 | **Exposure with response prevention (ERP):** Introduction to ERP and participants are presented with different strategies for conducting ERP. Examples are given of treatment goals and different ways of constructing exposure hierarchies. Participants construct an exposure hierarchy and goals for treatment. | 24 (6%) |
| 5 | **More on ERP:** Different aspects of ERP are highlighted, and a more in-depth explanation is given on how to work with ERP over time. | 22 (5%) |
| 6 | **Imaginal exposure:** Participants continue with ERP and are also introduced to imaginal exposure techniques. The participants are instructed to practice imaginal exposure for a certain obsessional thought. | 22 (5%) |
| 7 | **Re-exposure:** ERP exercises continues, and the participants learn techniques for re-exposure and are instructed to practice re-exposure. | 11 (3%) |
| 8 | **Difficulties during treatment:** Commonly encountered difficulties during treatment are presented and discussed. Examples are: loss of motivation and problems in integrating exercises into the daily schedule, common obstacles associated with ERP and how to overcome them. | 12 (3%) |
| 9 | **Values-based behaviour change:** Daily ERP continues with further exercises added that are adopted from acceptance and commitment therapy, including valued based goals and how they are applied in daily exposure tasks. | 23 (5%) |
| 10 | **Relapse prevention:** The treatment is summarised, and the participants are taught the distinction between relapse and setback. Participants construct their own relapse prevention program. | 264 (61%) |
|  | | |

**eMethod 3.**

|  | **BDD-NET: Description of module content and number of participants completing each module** |  |
| --- | --- | --- |
| 1 | **Psychoeducation:** Introduction to the treatment and information about BDD (prevalence, known etiology and common symptoms). Fictional patient characters are presented as examples to help clarify the treatment components throughout the treatment. Participants begin to register BDD-related behaviors and thoughts in an online diary | 3 (2%) |
| 2 | **A cognitive–behavioral conceptualization:** Explanation of how BDD-related avoidance and safety behaviors and self-defeating thoughts maintain appearance concerns and fears. Participants learn how to conduct a functional analysis of how their own BDD symptoms are maintained | 14  (9%) |
| 3 | **Cognitive restructuring:** A more in-depth explanation how self-defeating thoughts and maladaptive thinking maintain BDD symptoms. Participants evaluate negative thoughts and engage in cognitive restructuring using online worksheets | 5 (3%) |
| 4 | **Exposure with response prevention (ERP):** Explanation of ERP and different strategies for conducting response prevention. Participants set treatment goals and conduct their first in vivo ERP exercise. ERP continues during the remainder of treatment, and participants continuously assess the outcome of ERP using an online worksheet | 13 (8%) |
| 5 | **More on ERP:** Different aspects of ERP are highlighted, and a more in-depth explanation is given on how to work with ERP | 7 (4%) |
| 6 | **Values-based behavior change:** Participants identify values-based long-term goals in the domains of relationships, career and leisure activities. Participant are instructed to take on an accepting standpoint towards negative thoughts and experiences, as an alternative to attempts to control these experiences, and at the same time try to engage in meaningful values-based activities | 11 (7%) |
| 7 | **Difficulties during treatment:** Commonly encountered difficulties during treatment are presented and discussed. Examples are: loss of motivation and problems in integrating exercises into the daily schedule, common obstacles associated with ERP and how to overcome them | 9 (6%) |
| 8 | **Relapse prevention:** Participants are presented with strategies on how to handle relapses into repetitive behaviors and avoidance behaviors. Participants also summaries the main lessons learnt, their future plans and what has been gained through the treatment | 100 (61%) |
|  |  |  |

**eFigure 1.** **Online Treatment Platform, Homework view**


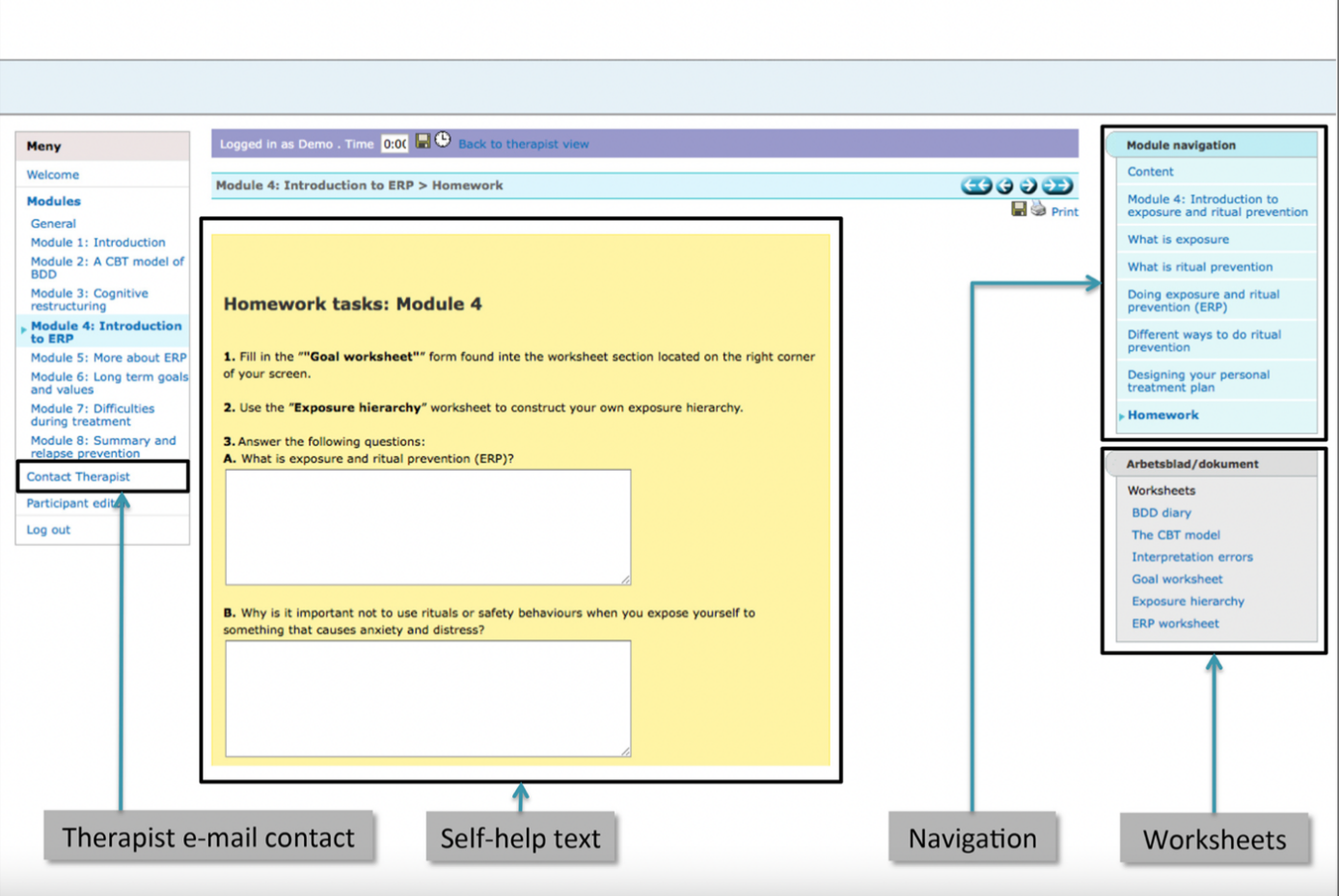


**eTable 1.** **Body areas of concern, BDD-NET**

| **Body areas of concern, N (%)** | **BDD-NET** |
| --- | --- |
| Skin | 52 (44) |
| Face | 45 (38) |
| Nose | 45 (38) |
| Hair | 37 (31) |
| Waist/Belly | 30 (25) |
| Eyes | 25 (21) |
| Chin | 22 (18) |
| Mouth | 20 (17) |
| Teeth | 18 (15) |
| Breast | 18 (15) |
| Arms/hands | 18 (15) |
| Head shape | 15 (13) |
| Cheeks | 14 (12) |
| Eyebrows | 12 (10) |
| Body hair | 7 (6) |
| Ears | 5 (4) |
| Muscles | 5 (4) |
| Gentiles | 3 (3) |
| All body areas of concern reported by the participants | |

**eTable 2.** **Results from missingness analysis**

To evaluate the mechanism of missingness, participants with and without Y-BOCS and BDD Y-BOCS post data were compared on the baseline variables: pre symptoms, age, gender, educational level, comorbidity and age onset for symptoms.

| **Baseline variable** | **OCD-NET (P-value)** | **BDD-NET (P-value)** |
| --- | --- | --- |
| Y-BOCS | 0.204 | N/A |
| BDD Y-BOCS | N/A | 0.372 |
| MADRS-S | 0.349 | 0.062 |
| Age | 0.754 | 0.395 |
| Gender | 0.576 | 0.001 |
| Educational level | 0.417 | 0.485 |
| Comorbidity | 0.851 | 0.189 |
| Age onset | 0.184 | 0.002 |
| Abbrivations: N/A = not applicable | | |

**eTable 3.** **Weekly results for the secondary outcome measures**

| **Outcome** | **Mean and (SE)** |
| --- | --- |
| **OCI-R** | |
| Pre | 22.3 (0.56) |
| Week 1 | 20.6 (0.56) |
| Week 2 | 19.7 (0.56) |
| Week 3 | 18.9 (0.56) |
| Week 4 | 18.1 (0.57) |
| Week 5 | 17.1 (0.57) |
| Week 6 | 16.5 (0.57) |
| Week 7 | 15.7 (0.57) |
| Week 8 | 14.8 (0.58) |
| Week 9 | 14.1 (0.58) |
| Week 10 | 13.5 (0.58) |
| Post | 13.3 (0.57) |
| **AAI** | |
| Pre | 30.6 (0.81) |
| Week 1 | 29.3 (0.82) |
| Week 2 | 27.7 (0.82) |
| Week 3 | 26.0 (0.83) |
| Week 4 | 24.3 (0.84) |
| Week 5 | 22.2 (0.84) |
| Week 6 | 22.0 (0.85) |
| Week 7 | 20.7 (0.86) |
| Week 8 | 19.8 (0.87) |
| Week 9 | 18.5 (0.87) |
| Week 10 | 17.7 (0.87) |
| Post | 17.5 (0.84) |
| **MADRS-S *(OCD-NET)*** | |
| Pre | 16.7 (0.43) |
| Week 1 | 15.4 (0.43) |
| Week 2 | 15.2 (0.43) |
| Week 3 | 14.6 (0.43) |
| Week 4 | 14.3 (0.44) |
| Week 5 | 13.9 (0.44) |
| Week 6 | 13.9 (0.45) |
| Week 7 | 13.5 (0.45) |
| Week 8 | 12.7 (0.45) |
| Week 9 | 12.1 (0.45) |
| Week 10 | 11.9 (0.45) |
| Post | 11.8 (0.44) |
| **MADRS-S (*BDD-NET)*** | |
| Pre | 19.0 (0.73) |
| Week 1 | 18.0 (0.73) |
| Week 2 | 17.8 (0.73) |
| Week 3 | 16.8 (0.74) |
| Week 4 | 16.2 (0.75) |
| Week 5 | 15.2 (0.75) |
| Week 6 | 15.9 (0.76) |
| Week 7 | 15.0 (0.77) |
| Week 8 | 15.0 (0.78) |
| Week 9 | 14.4 (0.77) |
| Week 10 | 13.3 (0.77) |
| Post | 12.6 (0.75) |
| Abbreviations: AAI, Appearance Anxiety Inventory; MADRS-S, Montgomery-Åsberg Depression Rating Scale Self-Rated; OCI-R, Obsessive-compulsive Inventory – Revised | |

**eTable 4. Results for the complete case analysis of response/remission**

Complete cases were participants providing both pre and post Y-BOCS and BDD-YBOCS data.

| **Treatment** | **Response (95% CL)** | **Remission (95% CL)** |
| --- | --- | --- |
| OCD-NET | 50 % (44% to 56%) | 22 % (17% to 28%) |
| BDD-NET | 68 % (57% to 77%) | 49 % (39% to 59%) |
| Abbrivations: CL, confidence interval; OCD-NET Response (Y-BOCS score reduction ≥ 35% and CGI-I ≤ 2) Remission (Y-BOCS score ≤ 12 and CGI-I ≤ 2)^14^; BDD-NET Response (BDD-YBOCS score reduction ≥ 30%) and Remission (BDD-YOCS score ≤ 16)^15^ | | |

**eTable 5.** **Results from the Negative effects questionnaire (NEQ)**

| **Item** | **OCD-NET** | **BDD-NET** |
| --- | --- | --- |
|  | **N (%)** | **N (%)** |
| **3: I experienced more anxiety** | 180 (52) | 65 (52) |
| **11: I experienced more unpleasant feelings** | 169 (50) | 62 (50) |
| **2: I felt like I was under more stress** | 153 (44) | 57 (45) |
| **13: Unpleasant memories resurfaced** | 110 (33) | 49 (39) |
| **4: I felt more worried** | 112 (32) | 40 (32) |
| **22: I did not always understand the treatment** | 81 (24) | 31 (25) |
| **5: I felt more dejected** | 65 (19) | 28 (22) |
| **18: I started thinking that the issue I was seeking help for could not be made any better** | 64 (19) | 33 (26) |
| **31: I felt that I was not emotionally touched by the treatment text** | 55 (16) | 41 (33) |
| **9: I felt sadder** | 53 (15) | 32 (25) |
| The table shows participants 10 most reported negative effects, attributed to the treatment. | | |

**eReferences**

1. López-Pina JA, Sánchez-Meca J, López-López JA, et al. The Yale–Brown Obsessive Compulsive Scale: A Reliability Generalization Meta-Analysis. *Assessment*. 2015;22(5):619-628. doi:10.1177/1073191114551954

2. Goodman WK, Price LH, Rasmussen SA, et al. The Yale-Brown Obsessive Compulsive Scale. I. Development, use, and reliability. *Arch Gen Psychiatry*. 1989;46(11):1006-1011. doi:10.1001/archpsyc.1989.01810110048007

3. Foa EB, Huppert JD, Leiberg S, et al. The Obsessive-Compulsive Inventory: Development and validation of a short version. *Psychol Assess*. 2002;14(4):485-496. doi:10.1037/1040-3590.14.4.485

4. Abramowitz JS, Deacon BJ. Psychometric properties and construct validity of the Obsessive–Compulsive Inventory—Revised: Replication and extension with a clinical sample. *J Anxiety Disord*. 2006;20(8):1016-1035. doi:10.1016/j.janxdis.2006.03.001

5. Veale D, Eshkevari E, Kanakam N, Ellison N, Costa A, Werner T. The Appearance Anxiety Inventory: Validation of a Process Measure in the Treatment of Body Dysmorphic Disorder. *Behav Cogn Psychother*. 2014;42(5):605-616. doi:10.1017/S1352465813000556

6. Svanborg P, Åsberg M. A new self-rating scale for depression and anxiety states based on the Comprehensive Psychopathological Rating Scale. *Acta Psychiatr Scand*. 1994;89(1):21-28. doi:10.1111/j.1600-0447.1994.tb01480.x

7. Ravens-Sieberer U, Wille N, Badia X, et al. Feasibility, reliability, and validity of the EQ-5D-Y: results from a multinational study. *Qual Life Res*. 2010;19(6):887-897. doi:10.1007/s11136-010-9649-x

8. Guy W. Clinical global impression scale. Published online January 1976.

9. Zaider TI, Heimberg RG, Fresco DM, Schneier FR, Liebowitz MR. Evaluation of the Clinical Global Impression Scale among individuals with social anxiety disorder. *Psychol Med*. 2003;33(4):611-622. doi:10.1017/S0033291703007414

10. Kadouri A, Corruble E, Falissard B. The improved Clinical Global Impression Scale (iCGI): development and validation in depression. *BMC Psychiatry*. 2007;7(1):7. doi:10.1186/1471-244X-7-7

11. Devilly GJ, Borkovec TD. Psychometric properties of the credibility/expectancy questionnaire. *J Behav Ther Exp Psychiatry*. 2000;31(2):73-86. doi:10.1016/S0005-7916(00)00012-4

12. Attkisson CC, Zwick R. The client satisfaction questionnaire. *Eval Program Plann*. 1982;5(3):233-237. doi:10.1016/0149-7189(82)90074-X

13. Rozental A, Kottorp A, Forsström D, et al. The Negative Effects Questionnaire: psychometric properties of an instrument for assessing negative effects in psychological treatments. *Behav Cogn Psychother*. 2019;47(5):559-572. doi:10.1017/S1352465819000018

14. Mataix-Cols D, de la Cruz LF, Nordsletten AE, Lenhard F, Isomura K, Simpson HB. Towards an international expert consensus for defining treatment response, remission, recovery and relapse in obsessive-compulsive disorder. *World Psychiatry*. 2016;15(1):80-81. doi:10.1002/wps.20299

15. Fernández de la Cruz L, Enander J, Rück C, et al. Empirically defining treatment response and remission in body dysmorphic disorder. *Psychol Med*. 2021;51(1):83-89. doi:10.1017/S0033291719003003
